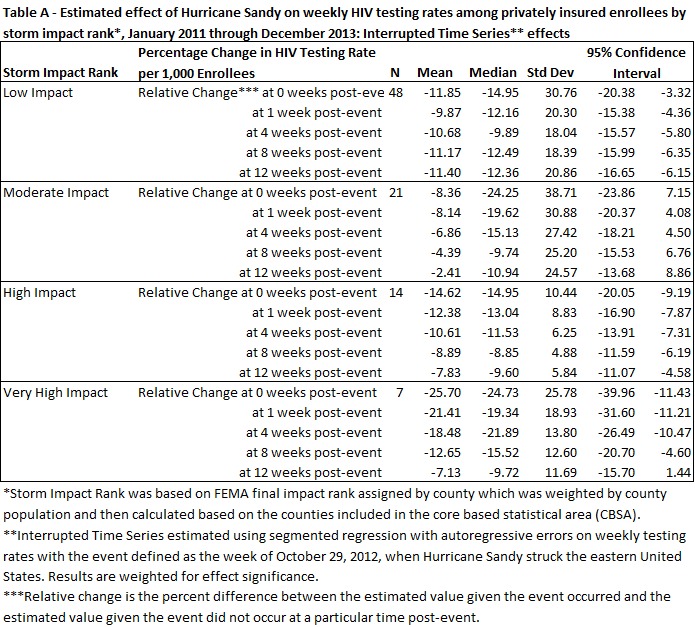# Vulnerabilities Associated with Post-disaster Declines in HIV-testing: Decomposing the Impact of Hurricane Sandy

**DOI:** 10.1371/currents.dis.e735c842bab99a2f564cc9a502394bbe

**Published:** 2018-08-21

**Authors:** Erin Thomas, Linda Ekperi, Tanya Telfair LeBlanc, Erica Elaine Adams, Grete E. Wilt, Noelle-Angelique Molinari, Eric G. Carbone

**Affiliations:** Centers for Disease Control and Prevention, Office of Public Health Preparedness and Response, Office of the Director, Office of Science and Public Health Practice (OSPHP); Centers for Disease Control and Prevention, Office of Public Health Preparedness and Response, Division of State and Local Readiness, Applied Science and Evaluation Branch (ASEB); Centers for Disease Control and Prevention, Office of Public Health Preparedness and Response, Division of State and Local Readiness, Applied Science and Evaluation Branch (ASEB); Centers for Disease Control and Prevention, Office of the Director, Division of Toxicology and Human Health Sciences, Geospatial Research, Analysis, and Services Program (GRASP); Centers for Disease Control and Prevention, Office of the Director, Division of Toxicology and Human Health Sciences, Geospatial Research, Analysis, and Services Program (GRASP); Centers for Disease Control and Prevention, Office of Public Health Preparedness and Response, Division of State and Local Readiness, Applied Science and Evaluation Branch (ASEB); Centers for Disease Control and Prevention, Office of Public Health Preparedness and Response, Office of the Director, Office of Science and Public Health Practice (OSPHP)

## Abstract

**Introduction:**

Using Interrupted Time Series Analysis and generalized estimating equations, this study identifies factors that influence the size and significance of Hurricane Sandy’s estimated impact on HIV testing in 90 core-based statistical areas from January 1, 2011 to December 31, 2013.

**Methods:**

Generalized estimating equations were used to examine the effects of sociodemographic and storm-related variables on relative change in HIV testing resulting from Interrupted Time Series analyses.

**Results:**

There is a significant negative relationship between HIV prevalence and the relative change in testing at all time periods. A one unit increase in HIV prevalence corresponds to a 35% decrease in relative testing the week of the storm and a 14% decrease in relative testing at week twelve. Building loss was also negatively associated with relative change for all time points. For example, a one unit increase in building loss at week 0 corresponds with an 8% decrease in the relative change in testing (p=0.0001) and a 2% at week twelve (p=0.001).

**Discussion:**

Our results demonstrate that HIV testing can be negatively affected during public health emergencies. Communities with high percentages of building loss and significant HIV disease burden should prioritize resumption of testing to support HIV prevention.

## Introduction

Emergency preparedness plans aim to anticipate and mitigate the direct and indirect effects of large scale disasters on the public’s health. Often, these plans deal with the direct impact of a natural disaster or its indirect effects on disease transmission. However, the prospect of natural disasters, such as severe weather events, also has broader implications for prevention strategies.

When Hurricane Sandy made landfall in October of 2012, the storm caused damage to homes and businesses, displaced survivors and crippled vital infrastructure systems such as power, transportation, water treatment and healthcare. 375, 000 housing units were destroyed in New Jersey and New York; 8.5 million people lost power across 21 states; and more than 1,000 patients had to be evacuated from metro area hospitals due to unsafe conditions such as untreated waste which flowed from the treatment plants and impacted the public water systems. 1^, ^2 At least sixty-seven people in the U.S. died as a direct result of the storm and many others faced infrastructure loss and other conditions that can affect disease prevention strategies and long-term health.^3^

The National HIV/AIDS Prevention Strategy identifies increasing the proportion of HIV-infected persons who know their status and linkage to care as targets for reducing the burden of the disease. Approximately 1.2 million people were living with HIV in the US in 2011. Among those, 14% had undiagnosed infection.^4^ Persons who are not aware of their HIV status contribute to a third of the ongoing transmission in the U.S.^5^ Diagnosis is a critical first step in managing HIV. Diagnosis helps to connect people to treatment, treatment can reduce viral load, increase immune function and reduce the risk of transmission. CDC recommends that adolescents and adults are tested for HIV at least once and that those with an increased risk for HIV infection are tested at least annually.^6^

Ekperi and colleagues^7^ have identified that Hurricane Sandy had a negative impact on HIV testing rates using Interrupted Time Series (ITS) analyses. Natural disasters impact individual, social and environmental contexts that can contribute to these testing declines. For example, disasters may increase individual risk behaviors (e.g., increase in sex risk and/or injection behaviors), lead to changes in sexual networks, result in population changes (e.g., migration), and impact a community’s self-efficacy. Disasters also impact a community’s ability to mobilize resources and result in infrastructure damage. These post-event changes pose a threat to established HIV testing and prevention strategies and can contribute to disease spread.^8^ Therefore, it is essential to examine the impact of this storm on HIV testing.

Pre-event factors can also impact testing declines. HIV testing is associated with age, gender, race, marital status and access to healthcare. For example, Merchant et al.^9^ found that individuals who were male, white, married, or never married/partnered and those with private insurance were more likely to have never been tested for HIV. Similarly, although having a provider recommend HIV testing increases the odds that someone will get tested,^10^ Anderson et. al.^11^ found that physician perceptions of a patient can affect a doctor’s likelihood of recommending HIV testing. Patients that are perceived to be lower risk (e.g., whites) are less likely to be referred for HIV testing.^11^ Taking these pre- and post-storm factors into account, this study identifies factors that influence the size and significance of the estimated impact that Hurricane Sandy had on HIV testing using data on storm and community characteristics. These impact estimates were produced using methods similar to those used in previous work 7 and are presented in the Appendix.

## Methods

**Data Sources**

Using Truven Health Analytics Commercial and Medicare supplemental Claims and Encounters Database, this study analyzed HIV testing rates in Sandy-affected regions from 2011 to 2013 by core based statistical area (CBSA). The database links paid claims and encounter data to detailed patient information across provider sites and types and comes from a selection of large employers, health plans, and government and public organizations.^12^ The database also contains person-specific clinical utilization and expenditures, enrollment for inpatient, outpatient, and prescription drug services, and services not covered in a health insurance contract.^12^ Interrupted Time Series (ITS) analyses, specified as segmented regressions with autoregressive errors, were estimated to capture the impact of Hurricane Sandy on weekly rates of HIV testing per 1,000 enrollees in CBSAs located within FEMA disaster-designated counties.

Weekly outpatient claims data from 2011 to 2013 were used to identify those who tested for HIV. Current Procedural Terminology (CPT) codes (‘86689’, ‘86701’, ‘86703’, ‘87389’, ‘87390’, ‘87534’, and ‘87535’) representing HIV testing procedures were extracted, along with enrollee ID and the enrollment detail information. Enrollment detail data contained demographic information by geographic location, age, and gender. The data captured for the region included all those who were tested and billed by private insurance companies in the states that were hit the hardest by Hurricane Sandy: Connecticut, Delaware, Indiana, Illinois, Kentucky, Maine, Maryland, Massachusetts, Michigan, New Hampshire, New Jersey, New York, North Carolina, Ohio, Pennsylvania, Rhode Island, Tennessee, Vermont, Virginia, Washington D.C. and West Virginia.

HIV testing rates were examined among enrollees not previously diagnosed with HIV to determine if Hurricane Sandy caused any disruption in HIV testing and relative and absolute effects were estimated using Interrupted Time Series (ITS) analyses detailed in the Appendix. Accordingly, the number of people tested for HIV was divided by the number of those enrolled members per capita in the affected CBSAs and multiplied by 1000. The HIV weekly testing rate was calculated as the number of HIV tests administered identified by CPT codes in each CBSA divided by the number of enrollees in the CBSA for each week in the study period. We used CBSAs due to the unavailability of county identifiers in the claims data, which led to the inability to accurately link storm-related factors to rural areas located outside CBSAs. Relative change due to the event was defined as the percentage difference between the estimated value given the event occurred and the estimated value given the event did not occur evaluated at a particular time after the event. . Note that the estimated effect is scaled to be relative to 1 with minimum of zero. Thus, a mean relative impact of 0.92 indicates an 8% decline in the testing rate given Hurricane Sandy occurred.

In order to estimate the effect of storm-related factors that contributed to HIV-testing declines, Federal Emergency Management Agency Modeling Task Force (FEMA MOTF) Hurricane Sandy impact analysis data on storm intensity (e.g., precipitation, buildings lost) by county was incorporated. The FEMA MOTF data are compiled by a task force of FEMA experts in hazard assessment and are used to develop estimates of impacts after events.^13^ These county-level data were crosswalked to CBSA by calculating the weighted mean levels of storm-related factors for each CBSA using data from the counties included in that CBSA and weighting by county population estimate. Rural areas were excluded because these areas are not included in CBSAs.

To estimate the impact of sociodemographic as well as healthcare access factors that may have contributed to HIV testing decline, Area Health Resource Files (AHRF) data were crosswalked from county to CBSA by calculating the weighted mean value for each CBSA using data from the counties included in that CBSA and weighting by county population estimate. AHRF include county, state and national files that offer a broad range of health resources and socioeconomic indicators, such as numbers of health facilities, health professionals, and health training programs and measures of resource scarcity. In addition, AHRF contains geographic codes and descriptors which enabled us to link the AHRF data to the claims and FEMA data.

**2.2 Measures**

Because AHRF data in particular provides many variables that aim to measure similar constructs, we ran simple bivariate correlations in order to begin assessing which to include in the model. Variables were selected based on the correlation results as well as prior evidence of significant impact based on literature review. Note that AHRF variables were only available for a subset of the 90 CBSAs included in the Sandy Impact Area. We tested a number of variables for inclusion in this model that were theoretically relevant. For example, income and per capita medical doctors (MDs) were tested but did not approach significance in any model. Percent minority and unemployment rate approached or reached significance at 0.05, so they were included in the model. Several additional measures, including precipitation, population exposed to storm surge, the natural log of median per capita income, hospital beds per capita, and MD’s per capita were also examined as independent predictors of HIV testing rate in our models but were not included due to lack of significance.

HIV prevalence. HIV prevalence rates among enrollees in each CBSA for 2012 were calculated using MarketScan data. The number of members with HIV diagnoses in a CBSA was divided by the total number of members with and without HIV per CBSA and multiplied by 100,000.

Household damage. Total count of households with damage claims after Hurricane Sandy was divided by the total number of households in that CBSA in 2010 – the nearest year for which data was available – and multiplied by 100 to generate percentage of households damaged. (FEMA data).

Building loss. FEMA data included percentage of buildings lost due to Hurricane Sandy.

Housing Unit Density per square mile. Housing unit density per square mile based on 2010 census data – the time period with available data that most closely corresponded to Hurricane Sandy. (AHRF data)

Percentage minority population. Percentage of nonwhite or Hispanic/Latinos individuals in the population. The original data source for this variable was the 2010 Census.

Unemployment rate. The unemployment rate for 2010 was linked from AHRF and is based on 2010 Census data.

**2.3 Statistical Methods**

Estimates of the impact of Hurricane Sandy on weekly HIV testing rates per 1,000 enrollees not previously diagnosed with HIV by CBSA were calculated from parameter estimates retained from ITS analyses for each of 90 CBSAs included in the Sandy impact area.^7^^, ^14 Truven Health Analytics (Marketscan) data were used during the period between January 1, 2011 and December 31, 2013 in the northeastern region of the United States. ITS analyses were specified as segmented regressions with autoregressive errors. Note that ITS is a very robust quasi-experimental method for estimating the impact of an event. 15 See Appendix for additional information on ITS analysis.

Generalized Estimating Equations

We utilized a robust Generalized Estimating Equations (GEE) regression model to examine the effect of the sociodemographic and storm-related measures in the FEMA data and AHRF files on the relative change in HIV testing rates provided by ITS analysis. We estimated two GEE regression models at each of the five time points post-event; 0 weeks, 1 week, 4 weeks, 8 weeks, and 12 weeks. In Model 1, we utilized only FEMA data and HIV prevalence rates because these variables were available for the full sample of 90 CBSAs. Model 1 regressed the relative change in CBSA-specific HIV testing at each time point based on HIV prevalence rates, the percentage of households damaged, and the percentage of buildings lost in order to identify and examine the relationships of explanatory variables in the model at each time period and across time periods.

In Model 2, we included the AHRF variables, which reduced the sample size to 75 CBSAs. Model 2 regressed the relative change in CBSA-specific HIV testing at each time point based on HIV prevalence rates, the percent of households damaged, the percent of buildings lost, housing density per square mile, the percent of minority population, and the unemployment rate.

## Results

Table 1 presents summary statistics for the dependent (HIV testing rate per 1000 enrollees not previously diagnosed with HIV by CBSA) and independent variables. Summary statistics of the estimated relative impact of Hurricane Sandy on HIV testing rates is presented for five time periods post-event. While the relative impact does decline across the five time periods, at week 12 the mean and median relative impact are still over 4%. The standard deviation of the relative impact declines across the time periods, indicating reduced variability in the relative impact across time periods.

In Model 1, building loss is consistently negatively related to relative impact across the five time periods. When housing unit density per square mile is added in Model 2, HIV prevalence rate is negatively and significantly associated with change in HIV testing rates for all time periods. The relationship between HIV prevalence and change in testing becomes weaker over time – ranging from -0.0045 (p=.001) at Week 0 to -0.0016 (p=0.013) at Week 12. Percentage of households with damage is significant at all time points except Week 12, but is positively associated with HIV testing. The strength of this relationship similarly declines over time. Unlike household damage, the percent of buildings lost has a negative and significant relationship with change in HIV testing. The strength of this relationship declines over time, but it remains significant (Estimate = -0.107; p = .001) at Week 12. Finally, housing unit density per square mile is positively and significantly associated with change in HIV testing rates at all time periods.

Table 2 presents the relative marginal effects of the storm-related and sociodemographic factors on the relative impact to HIV testing rates for each model. Model 1 presents the 90 CBSA results with FEMA variables and HIV prevalence. Marginal effects represent the relative change in the dependent variable associated with a marginal, or incremental, change in the independent variable. Building loss was the only variable in this model to show significance at the 0.05 level. For example, at the time of the event, a 1% increase in building loss corresponds to 2% decrease in the relative change in testing at time 0, (or 98% of the baseline level). A 1% increase in building loss likewise corresponds to a 1% decrease in relative testing at week 4 and week 8.

Model 2 (Table 2) depicts relative marginal effects with the AHRF variables included.

In model 2, there is a significant negative relationship between HIV prevalence and the relative change in testing at all time periods. A 1% increase in HIV prevalence corresponds to a 35% decrease in relative testing the week of the storm and 14% decrease in relative testing by week twelve. Building loss was also negatively associated with relative change for all time points except for week twelve. For example, a 1% increase in building loss at week 0 corresponds with an 8% decrease in the relative change in testing (p=0.0001). At week 12, 1% increase in building loss corresponds to a 2% decrease in the relative change in testing (p=0.001).

In model 2, percentage of household damage maintained a significant and positive relationship with the relative change in testing across all time periods except for week twelve. A 1% increase in the percentage of household damage resulted in a 5% (p<0.0001) increase in testing at week zero. Percentage of household damage was significant at each time point except for week 12. Housing unit density per square mile and unemployment rate were also positively and significantly associated with the relative change in testing at all time periods. Percentage minority population was the only variable that was not significant across any of the five time periods, though it did approach significance at week 12 (p=0.0519).

**Conclusion**

In this study we have identified the effects of storm and sociodemographic factors that most contributed to declines in HIV testing after Hurricane Sandy. Our findings suggest that areas with higher levels of building loss are likely to experience a sharper decline in testing. Similarly, areas with higher HIV prevalence rates are also more likely to experience significant declines in HIV testing. Percentage of household damage maintained a significant and positive relationship with the change in HIV testing. Pre-storm variables such as housing unit density and the unemployment rate in a CBSA were also significantly and positively associated with HIV testing. These associations and their directions are consistent with the literature mentioned previously and may be due to differences in physician and patient perceptions of risk based on race and income and on differences in the availability of and access to HIV testing services in more urban versus less urban CBSAs.9-11

## Discussion

This study also reveals an important distinction between household damage and building loss. Percentage of household damage – the total count of households with damage divided by the total number of households in a CBSA – was positively and significantly associated with change in HIV testing while percentage of buildings lost was negatively and significantly associated with change in HIV testing. This suggests that percentage of buildings lost (e.g., healthcare facility loss), but not household damage, may be a better measure of HIV testing service availability and correlates to the overall storm impact that can contribute to testing declines.

This study uses ITS, a robust modeling technique that controls for prior trends and seasonality while estimating the impact of an event. This study also expands on previous analysis of HIV testing rates after Hurricane Sandy to unpack the pre- and post-storm variables that most significantly impact HIV testing. While the one potential limitation of ITS is confounding by a co-occurring event, the authors were not able to identify any co-occurring event that might have confounded the estimated effect of Hurricane Sandy.

This analysis was limited to CBSAs which did not allow estimation of similar effects on HIV testing rates in rural areas. Future studies need to address the effects of hurricanes on HIV testing rates in rural areas as well. This analysis was also restricted to private claims data. Future studies need to incorporate Medicare and Medicaid claims to better understand the impact on the broader population. Finally, while this study focuses exclusively on HIV testing rates, it is likely that that many other types of primary care monitoring/testing may be affected by disasters. Future research needs to assess the impact of a disaster on other primary care prevention activities such as breast screening, immunizations, blood sugar and blood pressure monitoring.

## Appendix

Statistical Methods: Interrupted Time Series Analysis

Descriptive statistics of mean, median, standard deviation, and range were calculated among the 90 CBSAs for the HIV testing rate per 1,000 enrollees before and after the event date by storm impact rank. Each of the time series was examined visually and trend and seasonal statistics were analyzed. There were 153 weeks in each time series with 95 weeks pre-event and 58 weeks post-event. Statistical analyses was conducted using the statistical software package SAS v9.3.

In order to estimate the impact of Sandy on HIV testing rates, ITS models of weekly rates were estimated via maximum likelihood for each of 90 CBSAs in the Sandy-affected area. ITS models were specified as linear segmented regression with autoregressive errors. Time series were tested for stationarity via the augmented Dickey-Fuller test and autocorrelation via generalized Durbin-Watson tests. Autoregressive error models were used to address autocorrelation and seasonality where present (Wagner, et al. 2002; Jandoc, et al. 2015). Models were estimated in SAS using proc autoreg. with autoregressive error terms identified via backward elimination.

The ITS was modeled using the following linear segmented regression with autoregressive errors, which represents the baseline level and trend of the outcome variable before the event and changes in the level and trend after the event for each of the six outcomes:

(1)

Here, Yt represents the dependent variable, weekly HIV testing rate, at a point in time. Timet is the continuous variable representing time in weeks since the beginning of the study period. The intervention function (B2*event(t)+B3*timeafterevent(t)) was specified as a step function. Eventt is an indicator variable set to zero prior to the date of the event and becoming 1 the week of the event and for the duration of the time series after the event. Timeaftereventt is a continuous variable counting the days elapsed since the event at time t and set to zero prior to the event. The regression error, et, is comprised of a random error component as well as an autoregressive error component to adjust for autocorrelation and seasonality.

The estimated factual case is represented as:

(2)

The estimated counterfactual case is represented as:

(3)

Equation (2) was estimated for each CBSA in the Sandy Impact area. The parameter estimates from equation (2) were used to calculate equation (3) for each CBSA.

Note that the parameter estimate is the baseline level of the outcome variable at time zero, or the intercept. is the estimated baseline trend of the outcome variable, or the weekly deviation from the baseline level prior to the event. is the estimated absolute change in level, or intercept, of the HIV testing rate that occurs immediately following the event and is the estimated absolute change in trend that occurs after the event. The estimated relative, or percentage, change in the baseline level due to the event is , while the estimated relative change in trend is . Thus, is the estimated post-event baseline of the outcome variable and is the estimated post-event trend in the outcome variable. The estimated absolute impact of the event measured one week post-event is equation (2) minus equation (3) evaluated at t=97, or and the estimated relative impact of the event measured one week post-event is . Thus, absolute impact is the difference between the estimated values given the event occurred and the estimated value given the event did not occur at a particular time post-event. Relative impact is expressed as the percentage change in HIV testing rates in the factual case compared to the counterfactual case.

Estimates of the parameters and standard errors retrieved from equation (2) were used to produce estimates and associated 95% confidence intervals of the estimated relative changes in level and trend as well as the estimated relative impact of the event immediately and at one, four, eight, and twelve weeks post-event. Based on the work of Zhang, et al. 2009, the 95% confidence intervals of the estimated relative changes for baseline, trend, and the various time points post-event were calculated via the multivariate delta method. Specifically, parameter estimates from equation (2) for each CBSA were retrieved and used to estimate the expected value and the variance of the relative change at each time point post-event. Summary statistics for these estimated relative changes were calculated by storm impact rank at 0 weeks post-event, at 1 week post-event, at 4 weeks post-event, at 8 weeks post-event and at 12 weeks post-event and weighted for significance (Table A).

****Data Availability:****

All relevant data are reported in this manuscript. Data sources include Truven Health Analytics Commercial and Medicare supplemental Claims and Encounters Database, Federal Emergency Management Agency Modeling Task Force (FEMA MOTF) database and Area Health Resource Files (AHRF) data. AHRF and FEMA MOTF data are publically available and accessible. Data from Truven Health Analytics Commercial and Medicare Supplemental Claims and Encounters Database are designed to address the requirements of the Health Insurance Portability and Accountability Act of 1996 (HIPAA). The MarketScan Research Databases meet the criteria for a limited-use data set and contain none of the data elements prohibited by HIPAA for such data sets. Formal Data Use Agreements (DUAs) are in place with every entity that do not allow public disclosure of detailed datasets used in this study. The DUA’s are in place to protect both patient and hospital sensitive data from public disclosure as the data is classified as a “Limited Data Set” under the Health Insurance Portability and Accountability Act of 1996 (HIPAA) which is to be protected as personal health information (PHI). DUA’s only allow data sharing if it is aggregated and fully de-identified as presented in this paper. Posting detailed data online or providing access to the detailed data that allows replication of this study, or other use of the data to non-public health personnel constitutes a legal violation of these DUA’s.

For requests regarding this data, please contact:

Market Scan Information Systems, Inc.

815 Camarillo Springs rd. Suite B

Camarillo, CA. 93012

Phone: 800-MKT-SCAN (658-7226)

Fax: 855-MKT-SCAN (658-7226)

www.MarketScan.com

www.mDesking.com

**Conflicts of Interest:** None

**Corresponding Author:** Erin Thomas is the corresponding author for this article and can be reached via kqn4@cdc.gov.

**Estimated effort of Hurricane Sandy on weekly HIV testing rates among privately insured enrollees by storm impact rank*, January 2011 through December 2013: Interrupted Time Series** effects:**